# The evolutionary dynamics of viruses: virion release strategies, time delays and fitness minima

**DOI:** 10.1093/ve/veab039

**Published:** 2021-04-27

**Authors:** Jennifer S Lord, Michael B Bonsall

**Affiliations:** 1 Department of Vector Biology, Liverpool School of Tropical Medicine, Liverpool L3 5QA, UK; 2 Mathematical Ecology Research Group, Department of Zoology, University of Oxford, Oxford OX1 3SZ, UK

**Keywords:** invasion fitness, mathematical modeling, virus evolution

## Abstract

Viruses exhibit a diverse array of strategies for infecting host cells and for virion release after replication. Cell exit strategies generally involve either budding from the cell membrane or killing the host cell. The conditions under which either is at a selective advantage is a key question in the evolutionary theory of viruses, with the outcome having potentially important impacts on the course of infection and pathogenicity. Although a plethora of external factors will influence the fitness of either strategy; here, we focus just on the effects of the physical properties of the system. We develop theoretical approaches to assess the effects of the time delays between initial infection and virion release. We show that the length of the delay before apoptosis is an important trait in virus evolutionary dynamics. Our results show that for a fixed time to apoptosis, intermediate delays lead to virus fitness that is lower than short times to apoptosis — leading to an apoptotic strategy — and long times to apoptosis — leading to a budding strategy at the between-cell level. At fitness minima, selection is expected to be disruptive and the potential for adaptive radiation in virus strategies is feasible. Hence, the physical properties of the system are sufficient to explain the existence of both budding and virus-induced apoptosis. The fitness functions presented here provide a formal basis for further work focusing on the evolutionary implications of trade-offs between time delays, intracellular replication and resulting mutation rates.

## 1. Introduction

Viruses have evolved to infect a diverse range of hosts, from bacteria to vertebrates. For viruses infecting protozoan or animal cells, virions can exit infected cells either by crossing the cell membrane — herein referred to as budding — or by killing the cell (Freed 2004; Buchmann and Holmes 2015; Bird and Kirkegaard 2015). Leaving infected cells is the only way to infect new cells for many viruses. Those that are lysogenic, however, can be replicated along with host genetic material during cell division.

A key question in the evolutionary theory of viruses is under what conditions is budding, killing the host cell or lysogeny at a selective advantage? This question has been addressed in some detail for lytic and lysogenic phages (Stewart and Levin 1984; Bonachela and Levin 2014; Maslov and Sneppen 2015; Berngruber et al. 2015; Weitz et al. 2019; Li et al. 2020). In addition, both theoretical and experimental studies have considered the evolution of the duration of the latent period for phage — the time between infection and killing the cell (Abedon 1989; Wang et al. 1996; Abedon et al. 2001, 2003; Wang 2006; Chantranupong and Heineman 2012). Using optimality models, Abedon (1989), Wang et al. (1996) and Abedon et al. (2003) showed that although a longer latent period results in a higher yield, shorter latent periods may be selected for when host cell density is high. This is because at high cell densities, the phage latent periods are long relative to the time it takes to infect susceptible cells. Wang (2006) demonstrated experimentally that there is a linear association between the phage latent period and yield and that there is an intermediate optimal time to killing the host cell, but the specific timing differed from results obtained from modeling. Chantranupong and Heineman (2012) also showed discrepancies between theoretical predictions of the duration of the latent period and experimental results, suggesting that constraints and genetics affect the accuracy of model predictions. Nevertheless, these theoretical studies have provided a foundation for understanding the evolution of phage latent periods.

Most phages are transmitted either by killing the host cell or by lysogeny, but some can be secreted across the host envelope without killing the cell. As such, most of the theoretical work has focused on the former two strategies. However, viruses that infect animal or protozoan cells can either exit the cell by killing the host cell or by budding. This brings an extra dimension to the evolution of the latent period — should a virus inhibit cell death for as long as possible and exit cells by budding only? Few studies have addressed the evolution of virion release strategies for viruses other than phage. Some viruses that infect cells lacking a cell wall can also incorporate into host genetic material, but here we focus on budding and virus-induced cell death.

There are many ways viruses can control cell death (Hay and Kannourakis 2002), and the process of cell death itself varies (Fink and Cookson 2005). For simplicity, we refer to virus-induced cell death as apoptosis, to distinguish from background, or natural, cell death. Apoptosis is programmed, in contrast to necrosis, which is a passive, degenerative process (Fink and Cookson 2005). Viral components can either entirely prevent, delay, or induce apoptosis (Shen and Shenk 1995; Hardwick 1998; Hay and Kannourakis 2002; Everett and McFadden 2002). While apoptosis can be induced as a protective measure by the cell, a virus capable of rapid replication and release by inducing apoptosis may be at an advantage compared to a virus which inhibits apoptosis and exits cells by budding, if one way of preventing cell death is by restricting replication (Randall and Griffin 2017).

While virus-induced cell death is generally associated with non-enveloped viruses, such as picornaviruses, evidence shows that some non-enveloped virus-cell combinations can result in viral exit by traversing the cell membrane (Bird and Kirkegaard 2015). Furthermore, research involving single-cell analyses shows that both the cell and the virus can cause between-cell variation in time to apoptosis and virus yield. For example, 15 per cent to 30 per cent of poliovirus-infected cells failed to lyse, even at time points after twenty-four hours (Guo et al. 2017). Similarly, products of enveloped viruses can induce apoptosis, potentially to the advantage of the virus (Liao et al. 1997; Su et al. 2001).

Krakauer and Payne (1997) developed a differential equation model of between-cell virus transmission including both budding and apoptosis. The model was used to show that, in general, higher apoptosis rates will be selected for when the mean lifetime of the cell is high and the budding rate low. Their model assumed that budding begins immediately after cell infection and that the time to apoptosis is exponentially distributed.

Furthering work in this area for viruses of vertebrates, Komarova (2007) argued that differential efficiency of antibodies could explain the evolution of virion release by apoptosis. The theory was motivated by the assumption that budding and apoptotic viruses have similar intracellular replication rates and, in the absence of an antibody response, budding viruses that keep cells alive would have a selective advantage.

There is some evidence, however, that budding viruses have lower viral replication rates compared with apoptotic viruses. For example, Anderson et al. (1988) demonstrated that encapsidation of hepatitis A virus in cells inhibits transcription throughout the replication cycle, reducing overall virus production in comparison to other picornaviruses that cause cell death. For paramyxoviruses, Young et al. (2019) showed that single amino acid changes could convert an apoptotic to a budding infection by reducing intracellular viral replication at late stages of infection. Similarly, Frolov et al. (1999) suggested a direct correlation between viral RNA replication and cytopathogenicity for Sindbis virus.

In addition to variation in intracellular replication rates, the time to a virus either releasing mature virions by budding from a cell or the time to inducing apoptosis are likely two important parameters influencing the evolution of either strategy. The delay between cell infection and mature virion production is well documented, frequently referred to as the ‘eclipse phase’ (Davey et al. 1973; Uchil and Satchidanandam 2003; Baccam et al. 2006; Holder and Beauchemin 2011). Bonachela and Levin (2014) showed that modeling the latent period between infection and release as a fixed time delay, rather than exponentially distributed, affected evolutionary outcomes for phages, but to our knowledge similar theoretical studies for viruses capable of budding or causing apoptosis have not been carried out. There have been no attempts to consider the evolutionary dynamics of budding and apoptotic strategies, while accounting for potential differences in intracellular replication rates, alongside delays between infection and virion exit from cells.

Rather than focus on a single hypothesis — for example, antibody response — for the evolution of either strategy, here we look more broadly at virus evolutionary dynamics with respect to budding, apoptosis and the latent period. Considering evidence that viruses classically assumed to kill host cells may also exit by crossing the cell membrane and *vice versa* for viruses that predominantly bud (Liao et al. 1997; Su et al. 2001; Bird and Kirkegaard 2015), we develop theoretical approaches and determine: (1) the parameters most important in influencing virus evolutionary dynamics when both budding and apoptosis occur; (2) the impact of including a budding delay and fixed time to apoptosis on the relative fitness of apoptotic and budding strategies; and (3) the conditions under which either strategy is at a selective advantage.

## 2. Modeling between-cell virus transmission

### 2.1 Assuming constant hazard of apoptosis and immediate budding

We model virus infection of cells using the following three ordinary differential equations, with numbers of susceptible cells (S), infected cells (I) and virions (V) as state variables:

(1) $$\begin{array}{c} \frac{dS}{dt}=rS-\beta SV-\mu_{C}S\\ \frac{dI}{dt}=\beta SV-\mu_{C}I-\alpha I\\ \frac{dV}{dt}=\lambda I+\gamma \alpha I-\mu_{V}V \end{array}$$

where *r* is the cell replication rate, *β* is the virus infection rate on susceptible cells, *μ_C_* is the cell death rate, *λ* is the virus budding rate—the rate at which virions leave infected cells before cell death or apoptosis, *α* the apoptosis rate and *γ* the virus yield at apoptosis. Lastly, *μ_V_* is the virus decay rate.

At two extremes, if the budding rate (*λ*) is zero and the apoptosis rate (*α*) and virus yield at apoptosis (*γ*) are non-zero then the model reflects an apoptotic infection where virus kills the cell and virions are released only on apoptosis. If *α* and *γ* are zero and *λ* is non-zero, this reflects a budding infection with virions leaving the cell via budding only and virus not inducing apoptosis.

### 2.2 Assuming fixed time to apoptosis and budding delay

One simplifying, underlying assumption (in this model—Eq. 1) is that apoptosis is exponentially distributed and therefore could happen immediately after infection. By a similar assumption, new progeny virions can leave cells immediately by budding. This is violated in nature: there must be a period of RNA replication, protein production and genome encapsidation, before mature virions are produced (Regoes et al. 2005). We therefore replace dI/dt and dV/dt in the model in Eq. 1, to incorporate a fixed time to apoptosis (*τ*) and a time delay before virus budding can occur (τ′):

(2) $$\begin{array}{c} \frac{dI}{dt}=\beta SV-\mu_{C}I-\beta S(t-\tau )V(t-\tau )\;\text{exp}(-\mu_{C}\tau )\\ \frac{dV}{dt}=\lambda I(t-\tau \prime )\;\text{exp}(-\mu_{C}\tau \prime )+\gamma \beta S(t-\tau )V(t-\tau )\;\text{exp}(-\mu_{C}\tau )-\mu_{V}V. \end{array}$$

In this model, *τ* represents the time between a cell becoming infected and virus being released by apoptosis. The term $\beta S(t-\tau )V(t-\tau )\;\text{exp}(-\mu_{C}\tau )$ therefore represents the number of infected cells that have been infected for time *τ* and have not died from natural death (*μ_C_*). The case is similar for the terms including the virus budding rate (*λ*) and yield at apoptosis (*γ*).

**Figure 1.**
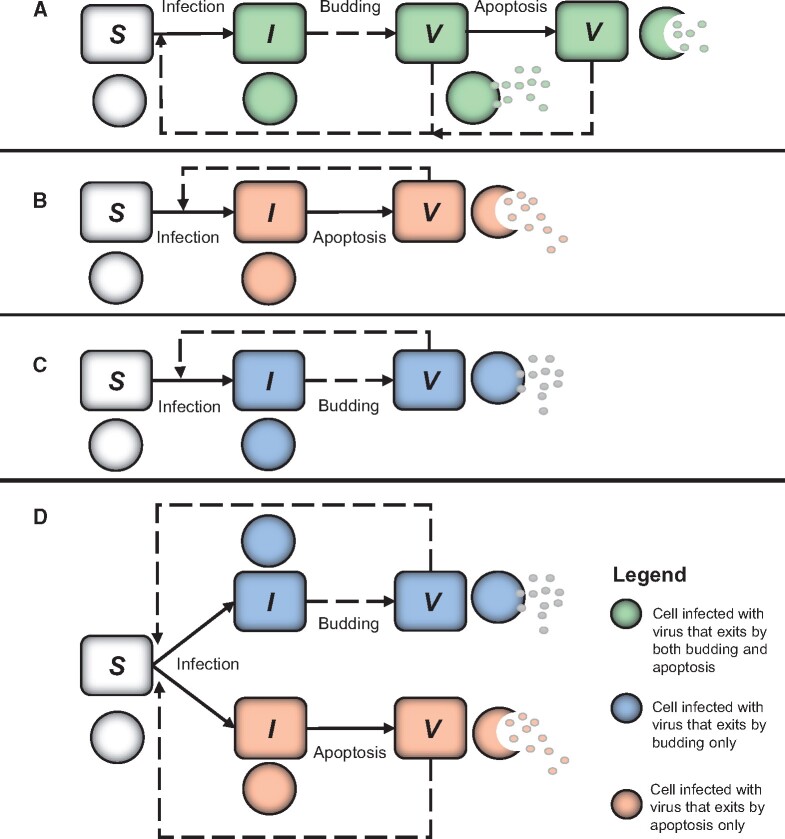
Schematic of models used to obtain virus fitness functions and in evolutionary invasion analysis. (A) The conceptual model for Eq. 1 (no delays) and Eq. 2 (delays). (B) The schematic for the model in Eq. 1 assuming the virus budding rate (*λ*) is zero and Supplementary Eq. S1 with no virus budding. (C) The schematic for the model in Eq. 1 assuming the apoptosis rate (*α*) and virus yield at apoptosis (*γ*) is zero and Supplementary Eq. S2 with no virus-induced apoptosis. (D) The models for budding only and apoptosis only strategies, combined in the evolutionary invasion analysis, where a resident virus that exits cells by budding is invaded by a mutant virus that exits cells by apoptosis (Eqs S15 and S16 of Supplementary Appendix A).

For the model without delays, we can set either the budding rate (*λ*) or the yield at apoptosis (*γ*) and the apoptosis rate (*α*), to zero to represent either of the virion release strategies. For the model including delays, variations of Eq. 2 are required to do this. Either the term including *λ* and τ′ is removed to represent a strategy where the virus kills the cell to release virions, or the terms involving the time to apoptosis (*τ*) and yield (*γ*) are removed to represent a purely budding strategy. These two model variations are provided in Supplementary Appendix A (Eqs. S1 and S2) and are used in the evolutionary invasion analysis to compare the two virus strategies, as described below. Figure 1A shows a schematic for the combined model and Fig. 1B and C shows the schematics for two separate models used in the evolutionary invasion analysis (Fig. 1D). The equilibrium for the number of susceptible cells is used in the evolutionary invasion analysis and the equilibrium conditions for all models are provided in Supplementary Appendix A (Eqs. S3–S6).

### 2.2.1 Virus fitness

Fitness is defined as the change in the *per capita* net growth rate (Fisher 1930; Michod 2000). Net growth rate is simply dX/dt and fitness is then (1/X)(dX/dt). For dX/dt=rX, fitness is *r*, the intrinsic rate of increase. Different mathematical approaches are required in deriving fitness functions (akin to the *per capita* net growth rate) when the underlying dynamics are more complex (Vincent and Brown 2005). The approach involves determining when the strategy can invade from rare and draws on the mathematics of dynamical systems theory. This approach has been widely used in deriving fitness functions for evolutionary ecological scenarios (Metz et al. 1992; Cohen et al. 1999; Bonsall and Mangel 2004, 2009; Klug and Bonsall 2014). Here, we show how this approach can be used to derive virus fitness functions from the governing equations for the virus dynamics.

Virus fitness is the outcome of virus infection, replication and survival. In our dynamical framework, these processes are considered completely; therefore, the resulting fitness functions consider the entire life cycle of the virus. In Supplementary Appendix A, we derive virus fitness functions for both models (Eqs. 1 and 2), and variations of the delay model with apoptosis (Supplementary Eq. S1) and budding only (Supplementary Eq. S2) strategies. The approach uses the determinant of a matrix of the partial derivatives of the contribution of infected cells (*I*) and free living virus (*V*), termed the Jacobian. The dominant eigenvalue of this matrix is a measure of virus fitness—equivalent to the *per capita* net growth rate. Note that this is not the same as the basic reproduction number. For simple systems, the equivalence of this interpretation with the basic reproduction number can be shown (Hurford et al. 2010). Positive fitness (positive eigenvalues) is required for virus to spread.

For the model without delays (Eq. 1), taking the determinant and setting equal to zero then solving the expression for *ω*, the eigenvalues of the matrix, gives a function for virus fitness (Supplementary Appendix A Eqs. S7–S9). Deriving the virus fitness functions for the models including fixed time to apoptosis and budding delay (Eq. 2 and Supplementary Eqs. S1–S2) is more complex. However, an approximation enables a function to be derived similar to that for the model without delays (Supplementary Appendix A S10–S12). We also use complex analysis to work through a full derivation of the invasion criteria to investigate the interplay between the time delays, budding rate and yield at apoptosis on virus fitness. This derivation is approached in a similar way to the simpler methods used to approximate virus fitness and is fully described in Supplementary Appendix A.

### 2.2.2 Evolutionary invasion analysis

The virus fitness functions as detailed in Supplementary Eqs. S7–S14 (Supplementary Appendix A) describe the intrinsic rate of increase for a single virus strategy. However, these fitness functions also provide, along with equilibrium conditions, the means to assess the ability of a mutant virus to invade a resident virus population and hence assess the relative fitness of two different virus strategies. An alternative mutant virus emerges from rare and competes with a resident virus. The competition between resident and mutant virus is mediated by the number of susceptible cells available for mutant virus to infect in the presence of the resident virus.

We assume that for a resident virus, the number of susceptible cells is at an equilibrium ($\hat{S}$), determined by the parameters of the resident virus. The other parameters for the fitness function are determined by the mutant virus—thus, the function describes the intrinsic rate of increase of a mutant virus if introduced to a resident virus infection at equilibrium. As the steady-state level of susceptible cells that the mutant virus experiences is set out in terms of the resident virus parameters, then locating the fitness boundaries in parameter space allows the effects of mutant virus evolution in the presence of resident virus to be investigated. This involves using numerical methods (see below) for solving these boundaries.

For both models, we investigate the conditions under which an apoptotic virus would be competitive against a budding virus. For this analysis, we assume that there is a resident virus capable of virion release by budding only and a mutant virus capable of virion release by apoptosis only. See Supplementary Appendix A for explanation of how this is derived from the models in Eq. 1 and Supplementary Eq. S1 (including budding delay) and Supplementary Eq. S2 (including fixed time to apoptosis). The resulting mutant virus fitness functions are given in Supplementary S15 and S16 (Supplementary Appendix A).

### 2.2.3 Numerical analyses

To quantify the effects of changes in model parameter values on virus fitness, we carried out thorough sensitivity analyses of the fitness functions. Latin hypercube sampling was used to generate 1,000 parameter sets for each function within the ranges provided in Table 1, assuming a uniform distribution for each parameter. Although estimates from the literature (Table 1) suggest that the delay between cell infection and apoptosis is frequently less than ten hours, longer times are used in sensitivity analyses to reflect a spectrum of viral strategies from early release of virions by apoptosis to predominant release by budding and keeping cells alive. For all analyses, the number of susceptible cells is $S=10^{6}$.

**Table 1.** Parameters and values used for sensitivity analysis.

Notation	Description	Value	Range	References
*β*	Probability of infection	10^-6^	0–10^-5^	
*λ*	Virus budding rate	100 (hours^−1^)	1–500	Poliovirus: Furness (1961) Semliki Forest and Kunjin virus: Davey et al. (1973); Japanese encephalitis virus: Uchil and Satchidanandam (2003); Vesicular stomatitis virus: Timm and Yin (2012)
*γ*	Virus yield at apoptosis	2400 (virions per cell)	1–12,000	Poliovirus: Furness (1961); Semliki Forest and Kunjin virus: Davey et al. (1973); Japanese encephalitis virus: Uchil and Satchidanandam (2003); Zika virus: Best et al. (2017)
*α*	Virus apoptosis rate	1/24 (hours^−1^)	1/200–1/2	Poliovirus: Furness (1961); Semliki Forest and Kunjin virus: Davey et al. (1973); Japanese encephalitis virus: Uchil and Satchidanandam (2003); Influenza virus: Holder and Beauchemin (2011); Vesicular stomatitis virus: Timm and Yin (2012); Zika virus: Best et al. (2017)
*τ*	Fixed time to apoptosis	24 (hours)	2–200	Poliovirus: Furness (1961); Semliki Forest and Kunjin virus: Davey et al. (1973); Japanese encephalitis virus: Uchil and Satchidanandam (2003); Influenza virus: Holder and Beauchemin (2011); Vesicular stomatitis virus: Timm and Yin (2012); Zika virus: Best et al. (2017)
τ′	Budding delay	2 (hours)	2–72	Poliovirus: Furness (1961); Semliki Forest and Kunjin virus: Davey et al. (1973); Japanese encephalitis virus: Uchil and Satchidanandam (2003); Influenza virus: Holder and Beauchemin (2011); Vesicular stomatitis virus: Timm and Yin (2012); Zika virus: Best et al. (2017)
*μ_V_*	Virus decay rate	0.1 (hours^−1^)	0.001–0.5	
*μ_C_*	Cell death rate	1/120 (hours^−1^)	1/500–1/24	

The value for *r* is not provided here as it does not feature in the virus fitness functions. Invasion analyses were carried out assuming that the system is at equilibrium.

For the evolutionary invasion analysis, we assume for both viruses $\beta =10^{-6}$ (probability of infection), $\mu_{V}=0.1\;\mathrm{hour}s^{-1}$ (virus clearance rate) and cell death rate—variable (*μ_C_*), were equivalent. We set *α* = 1/24 hours^−1^ (apoptosis rate) for the model without delays, and where appropriate, set τ = 24 hours (fixed time to apoptosis) and *τ*′ = 1 hour (budding delay) for the model with delays. The resident virus budding rate (*λ*) was set to 100 hours^−1^. The values for the virus yield at apoptosis (*γ*) obtained from the invasion analysis were divided by the average (1/*α*), or fixed (*τ*), time to apoptosis and subsequently by the resident virus budding rate (*λ*) to get a relative virion production rate necessary for invasion by an apoptotic virus.

## 3. Results

### 3.1 Virus fitness in the absence of delays

**Figure 2.**
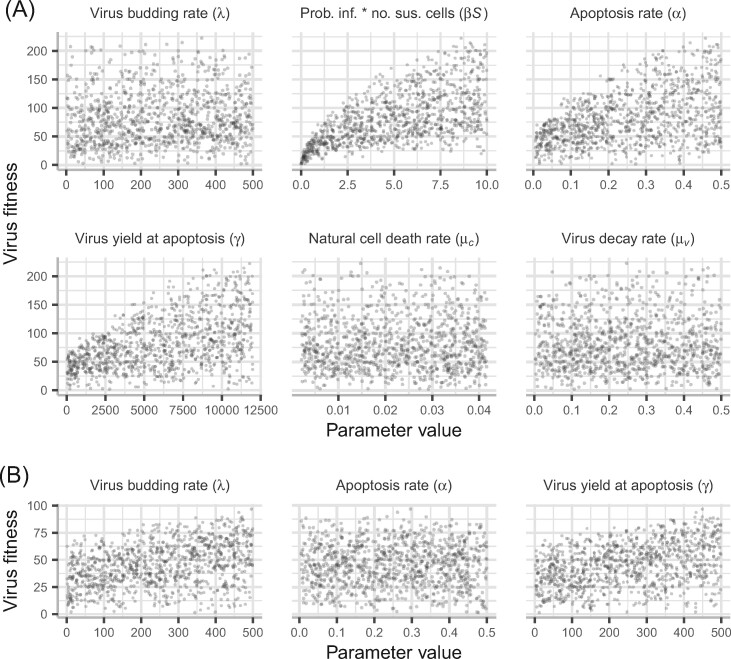
Virus fitness (Eq. S9, Supplementary Appendix A) for the model without delays (Eq. 1), as a function of each model parameter. Each plot shows virus fitness for 1,000 samples from each parameter range using Latin hypercube sampling and assuming a uniform distribution. Parameter ranges are detailed in Table 1. For the plots in (A) the virus yield at apoptosis (*γ*) is independent of the apoptosis rate (*α*), whereas for (B) (*γ*) scales with $\frac{1}{\alpha }$. See Table 1 for further details of the parameters.

For the model with immediate budding and a constant hazard of apoptosis (Eq. 1), virus fitness (Supplementary Eq. S9, Supplementary Appendix A) increases monotonically with the probability of infection (*β*), budding rate (*λ*), yield at apoptosis (*γ*) and the apoptosis rate (*α*), within the ranges given in Table 1 (Fig. 2). If the virus yield at apoptosis is independent of the apoptosis rate, fitness is particularly constrained by these two parameters, in addition to the probability of infection (Fig. 2A). For low values of the yield at apoptosis, average time to apoptosis (1/*α*) and the probability of infection, there is no combination of other parameter values, within the ranges used, that could result in a fitness equivalent to that achieved for higher values of these parameters. Conversely, relatively high fitness values could be obtained even when the budding rate (*λ*) is low (Fig. 2A). However, if virus yield at apoptosis (*γ*) increases as the apoptosis rate (*α*) decreases, assuming that the longer the cell is alive the more virions can be produced, fitness is no longer constrained by the apoptosis rate and the effects of the virus budding rate and yield at apoptosis are similar (Fig. 2B).

These results highlight that if both the virus yield and apoptosis rate can be maximised, there are conditions under which an apoptotic virus could be at an evolutionary advantage. In addition, assuming the probability of infection (*β*) and the virus decay rate (*μ_V_*) are equivalent between an apoptotic and budding virus, then the virus budding rate (*λ*), compared with the apoptosis rate (*α*) and yield at apoptosis (*γ*), relative to the cell death rate (*μ_C_*), will determine evolutionary outcomes.

**Figure 3.**
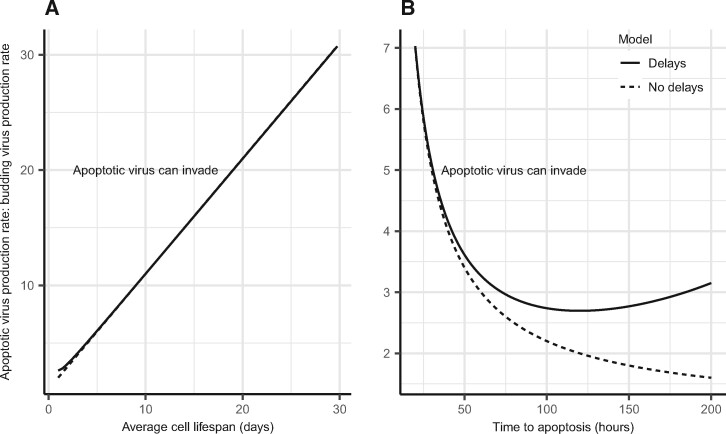
Evolutionary outcomes for apoptotic versus budding virus as a function of apoptotic virus production rate and: (A) average cell lifespan (*μ_C_*); (B) time to apoptosis ($\frac{1}{\alpha }$ for the model without delays or *τ* for the model with delays). Results determined by invasion analysis, assuming an apoptotic virus which releases virions by apoptosis invades a virus which releases virions by budding only. Lines represent equivalent fitness—above the line the apoptotic virus can invade, below the line the apoptotic virus cannot invade. The analysis assumed that the virus clearance rate *μ_V_* = 0.1 hours^−1^, probability of infection $\beta =10^{-6}$ and cell death rate *μ_C_* = 1/120 hours^−1^ (B) or variable (A), were equivalent for both viruses. For both plots, the budding delay τ′ = 1 hour for the resident virus. For (A), the mutant virus apoptosis rate α = 1/24 hours^−1^ for the model without delays and τ = 24 hours for the model with delays. For (B), the *x* axis represents the average time to apoptosis (1/α) for the model without delays and *τ* for the model with delays. The resident virus budding rate (*λ*) was arbitrarily set to 100 hours^−1^. We calculated the relative apoptotic intracellular virus production rate by dividing the resulting virus yield at apoptosis (*γ*) from the invasion analysis by the average or fixed time to apoptosis and then divided this by *λ*. Equations used to produce this figure are Eqs. S15 and S16 in Supplementary Appendix A.

Evolutionary invasion analysis shows a virus that only releases virions by apoptosis will be more competitive than a virus that only releases virions by budding, if its rate of intracellular virion production exceeds a given threshold. For example, if the cell death rate is 1/10 hours^−1^, the intracellular production rate of an apoptotic virus would need to be approximately ten times greater than that of a budding virus to invade, increasing linearly with the average cell lifespan (Fig. 3A). Similarly, as the average time to apoptosis increases, the virus yield at apoptosis would need to increase linearly for invasion to occur. However, the underlying rate of intracellular virion production required for an apoptotic virus to invade a resident budding virus would actually decline, under the assumption that yield is virus production rate per unit time multiplied by the total time to apoptosis (Fig. 3B). If the intracellular replication rate is equal between a budding and an apoptotic virus, the amount released upon apoptosis for the apoptotic virus will be lower than the total amount produced by a budding virus up until natural cell death of the persistently infected cell. In order for an apoptotic virus to be competitive, the intracellular rate of virus replication needs to only be sufficient to account for this discrepancy.

### 3.2 Virus fitness considering time delays

Including a budding delay and fixed time to apoptosis in the model (Eq. 2) gives similar results in terms of the relative amount of intracellular virion production an apoptotic virus would need, to be competitive against a budding virus, as a function of the cell death rate (Fig. 3A). For this model, however, the results of invasion analysis are not a linear function of the time to apoptosis (*τ*). The relative amount of virus produced per unit time by infected cells required for invasion would initially decline, but then increase (Fig. 3B).

**Figure 4:**
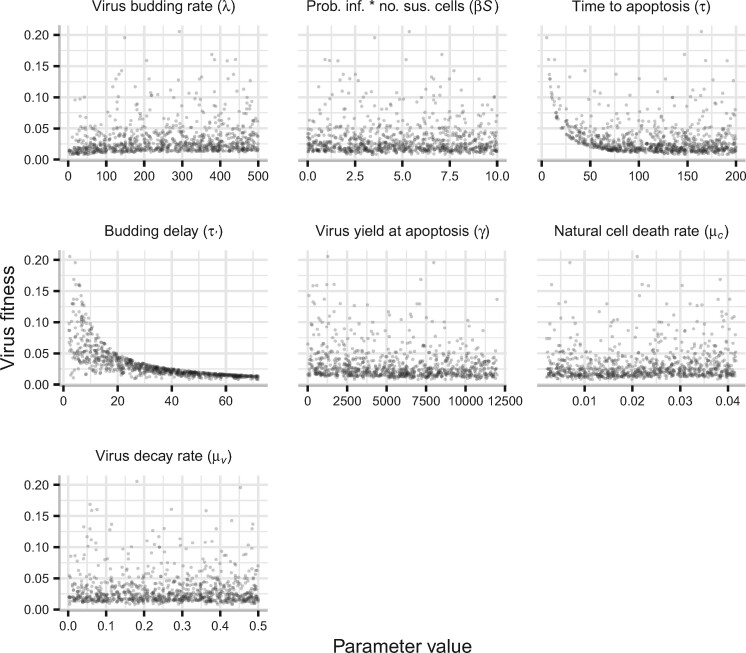
Virus fitness (Eq. S12, Supplementary Appendix A) for the model without delays (Eq. 2), as a function of each model parameter. Each plot shows virus fitness for 1,000 samples from each parameter range using Latin hypercube sampling and assuming a uniform distribution. Parameter ranges are detailed in Table 1. Samples where the budding delay was greater than the time to apoptosis (τ′ > τ) were omitted.

These differences are also reflected in the results of sensitivity analysis, where the budding delay (*τ*′) and time to apoptosis (*τ*) dominate the outcome of the virus fitness function relative to other parameter values (Fig. 4). In particular, virus fitness is constrained by the duration of the budding delay, whereas even for relatively long times to apoptosis, there are combinations of other parameter values that can lead to relatively high virus fitness (Fig. 4). By comparison of the plots in Figs. 2 and 4, it can be seen that the values for virus fitness are overall lower for the model including delays. This is because time delays affect survival—up to a point of invasion—and these losses accrue and therefore lower fitness relative to a system without delays. A simple example to illustrate this is shown in Supplementary Appendix A (Eqs. S17–S20).

**Figure 5:**
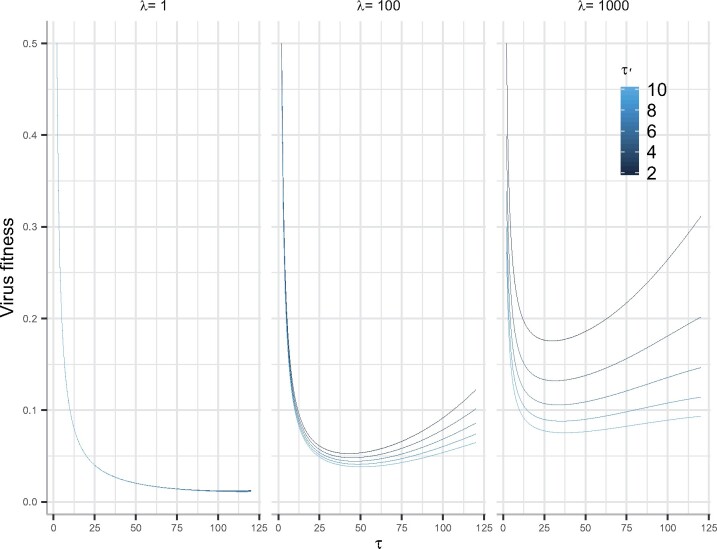
Virus fitness (Eq. S12, Supplementary Appendix A) for model including delays (Eq. 2) as a function of time to apoptosis (*τ*). Shorter budding delays (τ′), relative to average cell lifespan (*μ_C_*) and higher budding rates (*λ*) result in a fitness minimum. Other parameter values kept constant—background cell death rate (*μ_C_*) = 1/24 hours^−1^, virus clearance rate (*μ_V_*) = 0.1 hours^−1^, virus yield at apoptosis (*γ*) = 5,000, probability of susceptible cell infection multiplied by the number of infected cells (*βS*) = 1.

Fitness minima exist as a function of the time to apoptosis (*τ*) for some combinations of parameter values—particularly a short budding delay (*τ*′) relative to average cell lifespan (1*/μ_C_*) and a budding rate (*λ*) sufficient to contribute more to transmission as the apoptosis delay increases (Fig. 5).

To explore this fitness minimum further, and the interaction between the time delays (*τ*, τ′), yield at apoptosis (*γ*) and budding rate (*λ*), the full derivation of the virus invasion analysis (Eqs. S21–S28, Supplementary Appendix A) allows two different cases associated with different time delay constraints to be investigated. The first case is under conditions for long budding delays, where

(3) $$-ln\left[\frac{\mu_{C}\mu_{V}}{\gamma \beta S}\right]\frac{1}{\mu_{C}}>\tau$$

the relative ratio of virus births to deaths has to be greater than the apoptosis time delay (*τ*) for the virus to spread under long budding delays.

A second limiting case (Supplementary Appendix A) occurs when the apoptosis delay is long:

(4) $$-ln\left[\frac{\mu_{C}\mu_{V}}{\beta S\lambda }\right]\frac{1}{\mu_{C}}>\tau \prime$$

The relative ratio of virus births to deaths has to be greater than the budding time delay for the virus to spread under long apoptosis delays.

These limiting cases highlight that time lag differences in budding versus apoptosis can introduce trade-offs in virus fitness that influences the occurrence of fitness minima. The general invasion condition with explicit delays until virus budding and virus apoptosis is:

(5) $$\mu_{C}\gamma \;\text{exp}(-\mu_{C}\tau )+(1-\text{exp}(-\mu_{C}\tau ))\lambda \;\text{exp}(-\mu_{C}\tau \prime )>\left[\frac{\mu_{C}\mu_{V}}{\beta S}\right].$$

Other things being equal ($\mu_{V}=\mu_{C}=\beta S$), this expression can be simplified to:

(6) γ exp(−τ)+(1−exp(−τ))λ exp(−τ′)>1.

**Figure 6.**
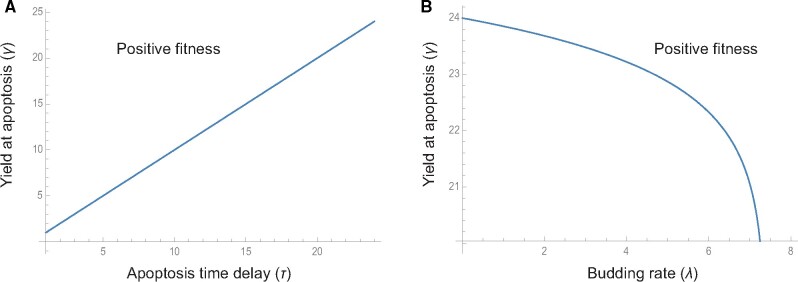
Trade-offs in investment in budding yield and yield at apoptosis. (A) Longer times (in hours) to apoptosis (*τ*) require greater investment in yield (parameters *λ*  =  0, τ′ = 2). (B) Other things being equal, differences in time delays generate trade-offs in budding yield—yield at apoptosis investment (parameters *τ*  =  24, τ′ = 2).

Solving this expression for the virus yield at apoptosis (*γ*) as a function of the virus budding rate (*λ*) shows that as the time to apoptosis increases, greater investment in virus yield (*γ*) is required to endure positive fitness (Fig. 6A). However, for fixed delays (τ′≤τ), as the yield from budding (*λ*) increases, less investment in virus yield at apoptosis is required to ensure positive fitness (Fig. 6B). This trade-off in investment emerges as a consequence of time-lag differences between budding and virus yield at apoptosis.

## 4. Discussion

Here, we have developed theoretical approaches to understand the interplay between apoptosis, budding and time delays on the evolution of virus replication strategies. Viruses cannot immediately leave host cells. Several steps of genome replication and assembly must be carried out before mature virions are produced. This results in a delay between infection and virus release. We have shown that the length of this delay is likely an important trait in virus evolutionary dynamics, for viruses that can either leave host cells by budding or killing the host cell.

Our results show that intermediate times to apoptosis lead to virus fitness that is lower than short times to apoptosis—leading to an apoptotic strategy—and long times to apoptosis—leading to a budding strategy at the between-cell level. At the between-cell level, trade-offs arise from the physical properties of the virus system. While the role of time delays on destabilizing dynamics in biological systems is well established (Mackey and Glass 1977; Gurney et al. 1980; Cooke and Grossman 1982), the evolutionary biological effects of explicit time lags seem less well developed (but see Fenton et al. 2006; Bonachela and Levin 2014). Here, we have shown how differences in time delays between virus budding and apoptosis are the explicit, physical drivers of trade-offs and hence lead to the formation of fitness minima in the adaptive landscapes (Fig. 4). At these minima, selection is expected to be disruptive and the potential for adaptive radiation in virus strategies is feasible. Understanding the potential for these trade-offs and time lags to generate multiple virus strains is beyond the scope of the current work but clearly a future next step in understanding the dynamics of virus evolutionary coexistence.

While there exists a body of theoretical work with respect to phage evolutionary dynamics (Stewart and Levin 1984; Bonachela and Levin 2014; Maslov and Sneppen 2015; Berngruber et al. 2015; Weitz et al. 2019; Li et al. 2020), including the evolution of phage lysis time (Abedon 1989; Wang et al. 1996; Abedon et al. 2001, 2003; Wang 2006; Chantranupong and Heineman 2012), there have been few mathematical analyses of evolution for viruses that do not undergo lysogeny and exit cells by either budding or apoptosis. We are only aware of two such studies (Krakauer and Payne 1997; Komarova 2007). For lytic phage, killing the host cell is the only way to release virions, and there is evidence that intermediate times are at an advantage (Wang 2006). This contrasts with our findings of fitness minima for intermediate times to apoptosis, for viruses able to exit cells also by budding.

Krakauer and Payne (1997) present a model similar to our first model with constant hazard of apoptosis and immediate budding, but used the levels of free virus or uninfected cells at equilibrium as a measure of fitness. Rather, our approach encompasses the entire virus life cycle in a single fitness function, as encouraged by Alizon and Michalakis (2015). Krakauer and Payne (1997) also assumed that virus could immediately start budding from infected cells. The analyses of our second model show that the budding delay is, however, likely an important parameter in virus evolutionary dynamics.

There are, of course, a plethora of external factors not accounted for in our relatively simple models of virus infection that will undoubtedly contribute to determining the relative fitness of either strategy in a given context. For viruses infecting multi-cellular organisms, cell type, in addition to immune responses, will be particularly important to consider. Infections of multi-cellular organisms therefore present a greater difficulty for modeling than chemostat systems of bacteria and phage. Our intention here was, however, to provide a general foundation for further work that would introduce trade-offs in the parameters in addition to the effects of external factors.

With respect to immunity, Komarova (2007) used a more complex model, including time delays and interactions with the immune system, to show that differential efficiency of antibodies could explain the evolution of virus release by killing host cells. While antibody responses of vertebrates may be an adequate hypothesis for the evolution of apoptotic viruses, here we have shown that a simpler explanation arises from the physical properties of the system.

While virus release by apoptosis may be at an advantage if apoptotic bodies containing virus go undetected by the immune system before they are taken up by susceptible cells (White 1996; O’Brien 1998), viruses that exit by budding may be able to transfer between adjacent cells, similarly avoiding the immune system (Bird and Kirkegaard 2015). As viruses have evolved a diverse range of strategies for evading host immune responses (Ploegh 1998), any future analyses that begin to incorporate these complexities will likely have to be tailored to specific virus and cell types, in contrast to our general approach here. Of relevance to our analysis is the ability of many viruses to inhibit, or postpone apoptosis, by targeting different cellular pathways, including those that counteract interferon (Ploegh 1998; Hay and Kannourakis 2002; Everett and McFadden 2002). The ability to postpone or completely inhibit apoptosis shows that viruses have evolved multiple strategies to alter the timing of cell death to their advantage. Our findings suggest that either times to virus production and release by apoptosis should be as short as possible, or relatively long to allow continued release of virus by budding.

Other, related, extensions to the analysis presented here would be to introduce trade-offs in the parameters that feature in the virus fitness functions, arising from intracellular replication dynamics. For example, an increase in the rate of intracellular replication can lead to earlier apoptosis (Frolov et al. 1999). While both trade-offs and external factors will likely influence the outcome of our analyses, it does not affect our conclusion that additional factors are not required to explain why both budding and apoptotic strategies exist.

Increases in the intracellular replication rate likely also have implications for mutation rates, leading to trade-offs in the amount of viable virus produced, the time to apoptosis as well as the evolutionary potential of a virus. For example, for positive-sense single-stranded RNA viruses, two extremes of virus replication within cells have been described and the effect on replication and mutation rates quantified (Thébaud et al. 2010; Sardanyés et al. 2012). Stamping machine replication is when all encapsidated viral genomes come from negative strands that are copies of the infecting genome. As there is only a single template within a cell, progeny viral genomes increase only linearly over time. Alternatively, geometric replication involves using multiple generations of positive strands as templates for the final genomes that become encapsidated. As a consequence, mutation rates will be higher for the geometric strategy and replication rate will be increased. Although few studies have estimated intracellular replication strategies, Martínez et al. (2011) demonstrated that turnip mosaic virus genomes arise from c. 93 per cent stamping machine. In contrast, Schulte et al. (2015) showed that poliovirus replicates predominantly by a geometric strategy.

Whether there is a general trend for apoptotic viruses to replicate geometrically remains to be quantified, but it provides a mechanistic explanation why some viruses can have higher intracellular replication rates, which may initiate cell death processes at earlier time points. The interplay between time delays, replication and mutation rates therefore have consequences for the evolutionary rates determined by different viral strategies. If apoptotic strategies arise because of geometric replication, an additional advantage may be generation of greater viral diversity and exploration of the fitness landscape.

The theoretical approaches developed here provide a formal definition of virus fitness at the cellular level and could be used to generate hypotheses and inform the design of *in vitro* experiments. For the evolutionary invasion analyses, we assume that the system is at a steady state before invasion by a mutant virus. This approach could be extended by relaxing the assumption that the system is at a steady state.

Our analysis has considered two extremes for modeling the virus within-cell latent period. We acknowledge that there is more likely to be an intermediate between these two models, with the time to budding or apoptosis varying between individual cells. However, our work serves as a basis for future analyses of infection strategies common to RNA viruses infecting multi-cellular organisms and similar to Bonachela and Levin (2014) for phages, has shown that model assumptions can have important implications for predicted evolutionary dynamics.

## Supplementary Material

veab039_Supplementary_Data
