# Supplementary material for: The evolutionary dynamics of viruses: virion release strategies, time delays and fitness minima
Source: Virus Evol. 2021 Apr 27;7(1):veab039. doi: 10.1093/ve/veab039 (PMC8242231; doi:10.1093/ve/veab039)
Supplement: veab039_Supplementary_Data [file veab039_supplementary_data.pdf]

Supplementary Appendix A

March 13, 2021

Delay model variations

Here we show the structure of two versions of the time-delayed model for the different assumptions about fixed time to apoptosis and budding delay.

Model with fixed time to apoptosis and no virus budding

$$\begin{aligned}\frac{dI}{dt} &= \beta SV - \mu_C I - \beta S(t-\tau)V(t-\tau)exp(-\mu_C\tau) \\ \frac{dV}{dt} &= \gamma\beta S(t-\tau)V(t-\tau)exp(-\mu_C\tau) - \mu_V V.\end{aligned} \tag{S1}$$

Model with budding delay and no virus-induced apoptosis

$$\begin{aligned}\frac{dI}{dt} &= \beta SV - \mu_C I \\ \frac{dV}{dt} &= \lambda I(t-\tau')exp(-\mu_C\tau') - \mu_V V.\end{aligned} \tag{S2}$$

The details of the parameters are given in the main text.

Equilibrium conditions

The equilibrium conditions for the model without delays (Eq. 1 in the main text) are:

$$\begin{aligned}\hat{S} &= \frac{\mu_V(\mu_C+\alpha)}{\beta(\alpha\gamma+\lambda)} \\ \hat{I} &= \frac{\mu_V(r-\mu_C)}{\beta(\alpha\gamma+\lambda)} \\ \hat{V} &= \frac{r-\mu_C}{\beta}\end{aligned} \tag{S3}$$

The equilibrium conditions for the model with delays (Eq. 2 in the main text) are:

$$\begin{aligned}\hat{S} &= \mu_V \left[\frac{\mu_C}{\lambda\beta(1-\sigma)\sigma'} + \frac{1}{\gamma\beta\sigma}\right] \\ \hat{I} &= \frac{\beta S^* V^*(1-\sigma)}{\mu_C} \\ \hat{V} &= \frac{r-\mu_C}{\beta}\end{aligned} \tag{S4}$$

where $\sigma = exp(-\mu_C\tau)$ and $\sigma' = exp(-\mu_C\tau')$ – the terms that account for natural cell death before either virus budding or virus release by apoptosis.

The equilibrium conditions for the model with fixed time to apoptosis and no virus budding (S1) are:

$$\begin{aligned}\hat{S} &= \frac{\mu_V}{\beta\gamma\sigma} \\ \hat{I} &= \frac{\beta S^* V^*(1-\sigma)}{\mu_C} \\ \hat{V} &= \frac{r-\mu_C}{\beta}\end{aligned} \tag{S5}$$

The equilibrium conditions for the model with budding delay and no apoptosis (S2) are:

$$\begin{aligned}\hat{S} &= \frac{\mu_C\mu_V}{\beta\lambda\sigma'} \\ \hat{I} &= \frac{\mu_V(r-\mu_C)}{\beta\lambda\sigma'} \\ \hat{V} &= \frac{r-\mu_C}{\beta}\end{aligned} \tag{S6}$$

Derivation of virus fitness functions

Model assuming constant hazard of apoptosis and immediate budding

The virus fitness function for the model without delays (Eq. 1 of the main manuscript) can be determined from the determinant of a matrix of the partial derivatives of the contribution of infected cells (I) and free living virus (V). This matrix ($\mathbf{J}$) has the form:

$$\mathbf{J} = \begin{pmatrix} -\omega-\mu_C-\alpha & \beta\hat{S} \\ \lambda+\gamma\alpha & -\omega-\mu_V \end{pmatrix} \tag{S7}$$

where ω are eigenvalues of the matrix (and the dominant eigenvalue is a measure of fitness). Taking the determinant of this matrix and setting equal to zero yields:

$$\omega^2 + \omega(\mu_C + \alpha + \mu_V) + \mu_V(\mu_C + \alpha) - \beta\hat{S}(\lambda + \gamma\alpha) = 0 \tag{S8}$$

Solving this expression for ω gives:

$$\omega = \frac{1}{2}\left(-(\alpha + \mu_C + \mu_V) \pm \sqrt{(\alpha + \mu_C + \mu_V)^2 - 4\left(\mu_V(\mu_C + \alpha) - \beta\hat{S}(\lambda + \gamma\alpha)\right)}\right). \tag{S9}$$

Virus fitness in the absence of delays is positive if the discriminant is greater than zero such that, $(\alpha + \mu_C + \mu_V)^2/4 > \left(\mu_V(\mu_C + \alpha) - \beta\hat{S}(\lambda + \gamma\alpha)\right)$ and this virus strategy will evolve.

Models with delays

A similar approach can be used to derive a fitness function for the virus model with fixed delays (Eq. 2 of the main manuscript). Due to the time delays, the matrix now has the form:

$$\mathbf{J} = \begin{pmatrix} -\omega - \mu_C & \beta\hat{S}\left(1 - exp(-\tau\omega)\sigma\right) \\ \lambda exp(-\omega\tau')\sigma' & -\omega - \mu_V + \gamma\beta\hat{S}exp(-\tau\omega)\sigma \end{pmatrix} \tag{S10}$$

where $\sigma = exp(-\mu_C\tau)$ and $\sigma' = exp(-\mu_C\tau')$. The determinant of this matrix is

$$\begin{aligned} &\omega^2 + \omega(\mu_C + \mu_V - \gamma\beta\hat{S}exp(-\tau\omega)\sigma) \\ &\quad + \mu_C(\mu_V - \gamma\beta\hat{S}exp(-\tau\omega)\sigma) - \lambda exp(-\omega\tau')\sigma'\beta\hat{S}\left(1 - exp(-\tau\omega)\sigma\right) = 0 \end{aligned} \tag{S11}$$

To solve this expression we begin by using $exp(-x) = 1 - x$ which is valid when x is small, under weak selection. This is a reasonable assumption if the difference between a mutant and a resident virus is small. The virus fitness function when fixed time to apoptosis and budding delay are included is:

$$\omega = \frac{1}{2(1+\beta\hat{S}(\gamma\sigma\tau+\lambda\sigma\sigma'\tau\tau'))}\Big(-\beta\hat{S}(\gamma\mu_C\sigma\tau-\gamma\sigma-\lambda\sigma\sigma'\tau-\lambda\sigma\sigma'\tau'+\lambda\sigma'\tau')-\mu_C-\mu_V$$
$$\pm\Big[-4(1+\beta\hat{S}(\gamma\sigma\tau+\lambda\sigma\sigma'\tau\tau'))(\mu_V\mu_C+\beta\hat{S}(\lambda\sigma\sigma'-\gamma\mu_C\sigma-\lambda\sigma'))$$
$$+(\beta\hat{S}(\gamma\mu_C\sigma\tau-\gamma\sigma-\lambda\sigma\sigma'\tau-\lambda\sigma\sigma'\tau'+\lambda\sigma'\tau')+\mu_C+\mu_V)^2\Big]^{1/2}\Big). \quad \text{(S12)}$$

In the same way as detailed above, we can derive fitness functions for the delay models with virus release from virus-induced apoptosis only (S1) and virus release by budding only (S2). With apoptosis only the fitness function is:

$$\omega = \frac{1}{2(1+\gamma\beta\hat{S}\tau\sigma)}\Big(-\beta\hat{S}\gamma\sigma(\mu_C\tau-1)-\mu_C-\mu_V$$
$$\pm\Big[-4(1+\gamma\beta\hat{S}\tau\sigma)(\mu_C(\mu_V-\gamma\beta\hat{S}\sigma))$$
$$+(\beta\hat{S}\gamma\sigma(\mu_C\tau-1)+\mu_C+\mu_V)^2\Big]^{1/2}\Big). \quad \text{(S13)}$$

With budding only (S2), the fitness function is:

$$\omega = \frac{1}{2}\Big(-\beta\hat{S}\lambda\sigma'\tau'-\mu_C-\mu_V\pm\Big[-4(\mu_C\mu_V-\beta\hat{S}\lambda\sigma')$$
$$+(\beta\hat{S}\lambda\sigma'\tau'+\mu_C+\mu_V)^2\Big]^{1/2}\Big). \quad \text{(S14)}$$

Evolutionary invasion analysis

Model assuming constant hazard of apoptosis and immediate budding

As described in the main text, we model the invasion, from rare of a 'mutant' virus (m) that releases virions by apoptosis, in the presence of a 'resident' virus that releases virions by budding. For the model assuming constant hazard of apoptosis and immediate budding, this uses the fitness function in Eq. S9, by setting the budding rate (λ) to zero and incorporating the equation for the steady state level of susceptible cells ($\hat{S}$) as per Eq. S3 where the apoptosis rate (α) and yield at apoptosis (γ) are set to zero, to give:

$$\omega = \frac{1}{2}(-\alpha_m-\mu_{Cm}-\mu_{Vm}\pm[-4\left(\mu_{Vm}(\mu_{Cm}+\alpha_m)-\gamma_m\alpha_m\beta_m\mu_V\mu_C/\beta\lambda\right)$$
$$+\left(\alpha_m+\mu_{Cm}+\mu_{Vm}\right)^2]^{1/2}). \quad \text{(S15)}$$

Models with delays

For the models with delays, we use the virus fitness function for the apoptosis only strategy (S13) and replace the steady-state levels of susceptible cells ($\hat{S}$) with the formula in Eq S6 for the budding only strategy to give:

$$\omega = \frac{1}{2(1+\gamma_m\beta_m\hat{S}\tau_m\sigma_m)}\Big(-\beta_m\hat{S}\gamma_m\sigma_m(\mu_{Cm}\tau_m-1)-\mu_{Cm}-\mu_{Vm} \pm \Big[-4(1+\gamma_m\beta_m\hat{S}\tau_m\sigma_m)(\mu_{Cm}(\mu_{Vm}-\gamma_m\beta_m\hat{S}\sigma_m)) + (\beta_m\hat{S}\gamma_m\sigma_m(\mu_{Cm}\tau_m-1)+\mu_{Cm}+\mu_{Vm})^2\Big]^{1/2}\Big) \quad \text{(S16)}$$

Where $\hat{S} = \mu_C\mu_V/\beta\lambda\sigma'$

Comparing fitness between models with and without delays

We can show using a simple example why values for fitness are generally lower for the model including delays. For the model without delays, if:

$$\frac{dV}{dt} = \beta SV - \mu_V V$$
$$\frac{1}{V}\frac{dV}{dt} = \beta S - \mu_V \quad \text{(S17)}$$
$$\text{(1)}$$

and we set $\beta = S = \mu_V = 1$, then virus fitness is:

$$\frac{1}{V}\frac{dV}{dt} = 1. \quad \text{(S18)}$$

However, with delays:

$$\frac{dV}{dt} = \beta SV exp(-\mu_C\tau) - \mu_V V \quad \text{(S19)}$$
$$\frac{1}{V}\frac{dV}{dt} = \beta S exp(-\mu_C\tau) - \mu_V$$

setting $\beta = S = \mu_V = 1$, then virus fitness is:

$$\frac{1}{V}\frac{dV}{dt} = exp(-\mu_C \tau) \tag{S20}$$

With time delays, fitness is expected to be reduced due to the inherent 'costs' associated with a delay.

Full derivation of the time delay model

We use the full derivation of the time delay model to investigate the interplay between the time delays, budding rate and yield at apoptosis on virus fitness. This derivation is approached in a similar way to the simpler methods used to approximate virus fitness. As above, we start from the point where the trivial steady state (when $V^* = 0$) is perturbed and now assume that the perturbation lasts from $t_0 - \tau$ to t_0, and if the displacement is $\delta\mathbf{x}(t)$, then, generally, $\mathbf{x}(t) = \mathbf{x} + \delta\mathbf{x}(t)$, and $d\mathbf{x}/dt = \mathbf{f}(\mathbf{x}(t)^*\delta\mathbf{x}, x^* + \delta\mathbf{x}(\mathbf{t} - \tau))$.

Given that $\mathbf{f}(\mathbf{x}^*, \mathbf{x}^*) = 0$, a Taylor series expansion of Eq. 2 in the main manuscript yields the following matrix:

$$\delta\mathbf{x} = \mathbf{J} = \begin{pmatrix} -\mu_C & \beta S\left[1 - exp(-\omega\tau)exp(-\mu_C\tau)\right] \\ \lambda exp(-\mu_C\tau')exp(-\omega\tau') & \gamma\beta S exp(-\omega\tau)exp(-\mu_C\tau) - \mu_V \end{pmatrix} \tag{S21}$$

In order to get nonzero solutions, we need that:

$$det(\mathbf{J} - \omega\mathbf{I}) = 0 \tag{S22}$$

where $\mathbf{I}$ is the identity matrix. This characteristic equation is then of the form:

$$\begin{aligned}\omega^2 &+ \omega\left(\mu_V - \gamma\chi + \mu_C\right) + \mu_C\mu_V - \mu_C\gamma\chi \\ &- \beta S\left(1 - exp(-\omega\tau)exp(-\mu_C\tau)\right)\lambda exp(-\mu_C\tau')exp(-\omega\tau') = 0\end{aligned} \tag{S23}$$

where $\chi = \beta S exp(-\omega\tau)exp(-\mu_C\tau)$. When $\omega = i\theta$ (as roots cross from the negative complex half-plane to the positive half-plane) then:

$$\begin{aligned}\theta^2 &+ i\theta(\mu_V + \gamma\chi + \mu_C) + \mu_C\mu_V - \mu_C\gamma\chi \\ &- \beta S\left(1 - \left[cos(\theta\tau) - isin(\theta\tau)\right]exp(-\mu_C\tau)\right)\lambda\left[cos(\theta\tau') - isin(\theta\tau')\right]exp(-\mu_C\tau') = 0\end{aligned} \tag{S24}$$

where $\chi = \beta S \left[cos(\theta\tau) - isin(\theta\tau) \right] exp(-\mu_C \tau)$. Equating real and imaginary parts yields the following set of simultaneous equations:

$$\theta^2 - \theta\gamma\beta S sin(\theta\tau) exp(-\mu_C\tau) + \mu_C\mu_V - \mu_C\gamma\beta S cos(\theta\tau) exp(-\mu_C\tau) \\ - \beta S(1 - cos(\theta\tau) exp(-\mu_C\tau))\lambda cos(\theta\tau') exp(-\mu_C\tau') = 0 \quad \text{(S25)}$$

$$\theta(\mu_V - \gamma\beta S cos(\theta\tau) exp(-\mu_C\tau) + \mu_C) - \mu_C\gamma\beta S sin(\theta\tau) exp(-\mu_C\tau) \\ - \beta S sin(\theta\tau) exp(-\mu_C\tau)\lambda sin(\theta\tau') exp(-\mu_C\tau') = 0. \quad \text{(S26)}$$

At the point of spread, when $\theta = 0$, then

$$\mu_C\mu_V - \mu_C\gamma\beta S exp(-\mu_C\tau) - \beta S(1 - exp(-\mu_C\tau))\lambda exp(-\mu_C\tau') = 0 \quad \text{(S27)}$$

So the the condition for the virus with budding and apoptosis to spread is:

$$\mu_C\gamma exp(-\mu_C\tau) + (1 - exp(-\mu_C\tau))\lambda exp(-\mu_C\tau') > [\mu_C\mu_V/\beta S] \quad \text{(S28)}$$

Using this approach we investigate the role of budding rate, yield at apoptosis and time delays on the evolution of virus strategies.

A limiting case - long budding delays

For long budding delays ($\tau' \to \infty$), $exp(-\mu_I\tau')exp(-\omega\tau') \to 0$, so the Jacobian is:

$$\delta\mathbf{x} = \mathbf{J} = \begin{pmatrix} -\mu_C & \beta S \left[1 - exp(-\omega\tau) exp(-\mu_C\tau)\right] \\ 0 & \gamma\beta S exp(-\omega\tau) exp(-\mu_C\tau) - \mu_V \end{pmatrix}$$

The characteristic equation is then:

$$\omega^2 + \omega\left(\mu_V - \gamma\beta S exp(-\omega\tau) exp(-\mu_C\tau) + \mu_C\right) \\ + \mu_C\left(\mu_V - \gamma\beta S exp(-\omega\tau) exp(-\mu_C\tau)\right) = 0$$

When $\omega = i\theta$ (as roots cross from the negative complex half-plane to the positive half-plane) then:

$$\theta^2 + i\theta\left(\mu_V - \gamma\beta S\left[cos(\theta\tau) - isin(\theta\tau)\right]exp(-\mu_C\tau) + \mu_C\right) \\ + \mu_C\left(\mu_V - \gamma\beta S\left[cos(\theta\tau) - isin(\theta\tau)\right]exp(-\mu_C\tau)\right) = 0$$

Equating real and imaginary parts yields the following set of simultaneous equations:

$$\theta^2 - \theta sin(\theta\tau) + \mu_C\left(\mu_V - \gamma\beta S cos(\theta\tau)exp(-\mu_C\tau)\right) = 0$$

$$\theta\left(\mu_V - \gamma\beta S cos(\theta\tau)exp(-\mu_C\tau) + \mu_C\right) - \mu_C\gamma\beta S sin(\theta\tau)exp(-\mu_C\tau) = 0.$$

If $\theta = 0$, then

$$\mu_C\left(\mu_V - \gamma\beta S exp(-\mu_C\tau)\right) = 0$$

$$-ln\left[\frac{\mu_C\mu_V}{\gamma\beta S}\right]\frac{1}{\mu_C} > \tau$$

A limiting case - long apoptosis delays

$$\delta\mathbf{x} = \mathbf{J} = \begin{pmatrix} -\mu_C & \beta S \\ \lambda exp(-\mu_C\tau')exp(-\omega\tau') & -\mu_V \end{pmatrix}$$

The characteristic equation is then:

$$\omega^2 + \omega(\mu_C + \mu_V) + \mu_C\mu_V - \beta S\lambda exp(-\mu_C\tau')exp(-\omega\tau') = 0$$

When $\omega = i\theta$ (as roots cross from the negative complex half-plane to the positive half-plane) then:

$$\theta^2 + i\theta(\mu_C + \mu_V) + \mu_C\mu_V - \beta S\lambda exp(-\mu_C\tau')\left[cos(\theta\tau') - isin(\theta\tau')\right] = 0$$

Equating real and imaginary parts yields the following set of simultaneous equations:

$$\theta^2 + \mu_C\mu_V - \beta S\lambda exp(-\mu_I\tau')cos(\theta\tau') = 0$$

$$\theta(\mu_C + \mu_V) + \beta S\lambda exp(-\mu_I\tau')sin(\theta\tau') = 0$$

If $\theta = 0$, then

$$\mu_C \mu_V - \beta S \lambda exp(-\mu_C \tau') = 0$$

$$-ln \left[\frac{\mu_C \mu_V}{\beta S \lambda} \right] \frac{1}{\mu_C} > \tau'$$

The relative ratio of virus births to deaths has to be greater than the budding time delay for the virus to spread under long apoptosis delays.
